# The fundamentals of eye tracking part 5: The importance of piloting

**DOI:** 10.3758/s13428-025-02737-9

**Published:** 2025-07-02

**Authors:** Roy S. Hessels, Diederick C. Niehorster, Marcus Nyström, Richard Andersson, Gijs A. Holleman, Ignace T. C. Hooge

**Affiliations:** 1 Experimental Psychology, Helmholtz Institute, Utrecht University, Heidelberglaan 1, 3584CS Utrecht, The Netherlands; 2 Lund University Humanities Lab, Lund, Sweden; 3 Department of Psychology, Lund University, Lund, Sweden; 4 Tobii AB, Danderyd, Sweden; 5 Department of Cognitive Neuropsychology, Tilburg University, Tilburg, The Netherlands

**Keywords:** Eye tracking, Eye movements, Pilot studies, Empirical cycle, Experiment design

## Abstract

The goal of this article is to demonstrate the importance of pilot studies in empirical eye-tracking research. First, we show what can go wrong when proper pilot experiments are omitted for all phases of an eye-tracking study, from testing an experiment, conducting the data collection, to building, revising, and interpreting the data analysis. Second, we describe a series of eye-tracking studies as a case study, and elaborate on all the pilot experiments that were conducted. We highlight what was learned from each pilot experiment when conceiving, designing, and conducting the research. Finally, we give practical advice for eye-tracking researchers on planning and conducting pilot experiments. This advice can be summarized as (1) take enough time, (2) be problem-oriented, (3) pilots are of an iterative nature, (4) many questions are empirical, and (5) apply the four-eyes principle. We envision that the present article helps early career researchers discover, and more established researchers rediscover, the utility of pilot experiments.

This article is the fifth installment in a series on the fundamentals of eye tracking. The articles are aimed at individuals who are (one of) the first in their group, company, or research field to use eye tracking, with a focus on all the decisions one may make in the context of an eye-tracking study. Such individuals may come from academia (e.g., psychology, biology, medicine, educational science, computer science), commercial institutions (e.g., marketing research, usability, decision-making) and non-commercial institutions (e.g., hospitals, air traffic control, military organizations). Note that this is not an exhaustive description of the target audience. More experienced eye-tracking researchers may find useful insights in the article series, or may find the article series a useful reference or hub to relevant research. Previous papers in the series have dealt with (1) the relation between theory and research question (Hessels et al., 2025b), (2) operationalizing research questions (Hooge et al., 2025), (3) choosing an eye tracker (Nyström et al., 2025), and (4) tools for eye tracking studies (Niehorster et al., 2025b). One may either start the series by reading the present article or first read the earlier articles in the series. This article discusses the importance of pilot studies in empirical eye-tracking research.

**Fig. 1:**
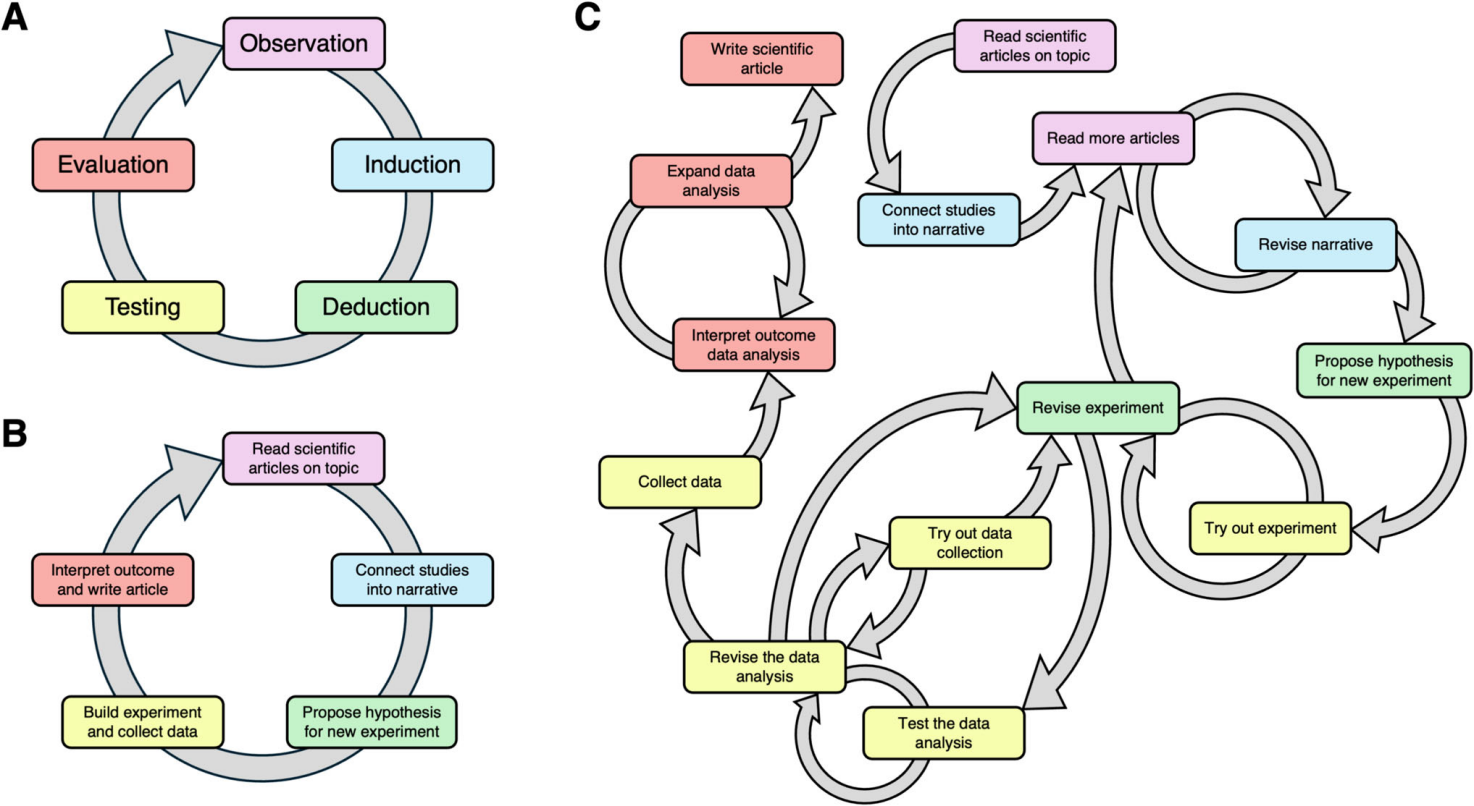
The empirical cycle. **A** The empirical cycle as proposed by de Groot (1961). **B** Naive application of the empirical cycle to empirical research. **C** One example of empirical reality; empirical cycles at multiple scales

Pick any introductory textbook in empirical (social) sciences or on research methods and one is likely to find a reference to the empirical cycle, which can be considered as a fundamental description of the practice of empirical research. The foundational text on the empirical cycle is by de Groot (1961) and it has proved consequential in the history of empirical science (see, e.g., Wagenmakers et al., 2018). For example, De Groot’s empirical cycle has been the basis for derivations in the context of clinical practice or design science (Van Strien, 1997; Wieringa, 2009), and it is utilized in the context of modern approaches to exploratory research (Van Lissa, 2023). De Groot’s empirical cycle consists of several phases that are run through in succession, including observation, induction, deduction, testing, and evaluation (see Fig. 1A). While it is hardly controversial that empirical science may be characterized along these phases, we have also seen the empirical cycle applied rather naively, for example by students or researchers new to empirical science. Such a more naive characterization can be given along the phases depicted in Fig. 1B. A single empirical study is seen as consisting of reading up on the scientific literature on a topic (observation), integrating these articles into a coherent narrative (induction), proposing a hypothesis and experiment to test this hypothesis (deduction), building an experiment and collecting data (testing), and finally the interpretation of the outcome and the production of a scientific paper (evaluation). In this naive characterization, *one* iteration of the cycle is almost synonymously used with the process of conducting and writing up an empirical paper^1^.

In our view, the naive characterization of the empirical cycle in Fig. 1B, glosses over two important points, already addressed in the original work by de Groot (1961). The first is that empirical cycles may occur at many different scales, within and between phases and with larger cycles potentially encompassing multiple smaller cycles (cf. section 1;2;3 in de Groot, 1961). The second is that preparatory (henceforth ‘pilot’) studies are crucial to empirical research: “... for good social-scientific research – as a rough estimate – about a quarter of the time, effort, and budget goes into preparation of the (testing-)setup” (de Groot, 1961, section 5;1;4, p. 146)^2^. In our view, Fig. 1C, is a more realistic depiction of empirical reality. Many shorter empirical cycles may be completed in the context of conducting an empirical study, with various ‘regressions’ to earlier phases in De Groot’s empirical cycle. For example, conducting an experiment may lead to new questions or lines of thought that can be explored in the existing scientific literature, or testing one’s data analysis may prompt a revision of the experiment (and possibly the hypothesis) altogether. It may even be the case that by the time one is drafting a scientific article, other articles have been published that report findings with potential consequences for the validity or interpretation of one’s experiment. In other words, ‘regressions’ to earlier phases of De Groot’s empirical cycle may be possible at every phase and with varying depth of regression.

We are keenly aware that not all students of social sciences, nor all researchers from non-empirical fields think along the lines of the naive empirical cycle just characterized. One may even claim that we are setting up a straw man argument. We would disagree. For example, we have even heard students of empirical research broaden claims of ‘not looking at your data before formulating your statistical hypothesis’ (learned in the context of statistical analysis) to apply to an entire study: If you conduct pilot experiments, you may effectively be ‘looking at your data’ or ‘hacking’ your way to a good outcome, which is not scientific. We disagree wholeheartedly with such an interpretation. More importantly, we believe that pilot studies or preparatory work are underappreciated in many research fields where eye tracking is used as a research method. Although many may answer affirmatively when asked about the importance of the pilot study, we observe surprisingly little piloting activity. That pilot studies may be underappreciated has also been suggested for other research contexts including, e.g., nursing research, clinical trials, and qualitative research (Prescott & Soeken, 1989; Thabane et al., 2010; Malmqvist et al., 2019). Moreover, that pilot studies are under-discussed is also evident from recent undergraduate textbooks on research methods in behavioral science. Gravetter and Forzano (2016), for example, do not mention pilot studies at all, while Cozby and Bates (2015) characterize pilot studies as having a narrow focus: “When the researcher has finally decided on all the specific aspects of the procedure, it is possible to conduct a pilot study in which the researcher does a trial run with a small number of participants” (p. 194). Thus, even if one does not recognize the naive characterization of the empirical cycle above, the importance of pilot experiments for empirical science is a topic worthy of discussion.

At the same time, we want to acknowledge that many researchers and students do appreciate the value of thoughtful piloting and apply it carefully within an empirical framework. Indeed, excellent examples exist where pilot work is used to validate methods or optimize the study design (see, e.g., Kumle et al., 2025). In line with many current open science practices or guidelines, piloting may help to determine the experiments or final analysis plan to submit in a preregistration, rather than changing analyses later after the main experiment is conducted. Moreover, pilot data can provide valuable input for power analyses, even for complex statistical models, at least when possible biases and appropriate corrections are considered (Albers & Lakens, 2018; Kumle et al., 2021). It is important to explicitly recognize that piloting may serve multiple roles within a confirmatory–exploratory framework: Pilot studies can help researchers develop robust, testable hypotheses and fine-tune experimental procedures. The main distinction with the idea that pilot experiments can be used to ‘hack’ one’s way to a good outcome, lies in maintaining the independence of exploration and testing specific hypotheses. Thus, rather than undermining scientific rigor, carefully planned and reported pilot work may strengthen methodological quality.

In this article, we aim to convince you, the reader, that even if you think you know that pilot experiments are important, they will save you more time and energy than you think. We will also bring up our own examples of costly mistakes that could have been avoided by pilot experiments. While there are existing articles on the uses of pilot studies, these are in the context of, for example, questionnaire validation (Van Teijlingen & Hundley, 2001) or randomized controlled trials (Lancaster et al., 2004; Thabane et al., 2010). We are not aware of articles dedicated to pilots in the context of eye-tracking research (although see Hessels et al., 2022; Fu et al., 2024, for some practical considerations). From a didactic standpoint, we believe it is crucial to situate the problem – how to use pilot experiments effectively and efficiently – in the specific research context, namely eye-tracking research. To achieve this, we will first show what can go wrong when conducting proper pilot experiments is overlooked for all phases of an eye-tracking study, from testing an experiment, conducting the data collection, to building, revising, and interpreting the data analysis. Second, we will consider a series of eye-tracking studies and elaborate on all the pilot experiments conducted when conceiving, designing, and conducting the research, while highlighting what we learned from each pilot. Finally, we end with practical advice for eye-tracking researchers on planning and conducting pilot experiments. Prior to this, however, we take some inspiration from art and product design to show the broad utility of a piloting mindset.

## A piloting mindset: Art, product design, and science

Mastery often relies on practice, which applies to professions from athletes to teachers and radiologists. But for artists, product designers, and scientists, such practice carries a unique twist: they make original works of art, create innovative products, or deliver scientific breakthroughs. These examples are characterized by delivering something novel each time. How are such novel creations achieved? For both art and product design, often not in one go, but rather through an iterative process.

**Fig. 2:**
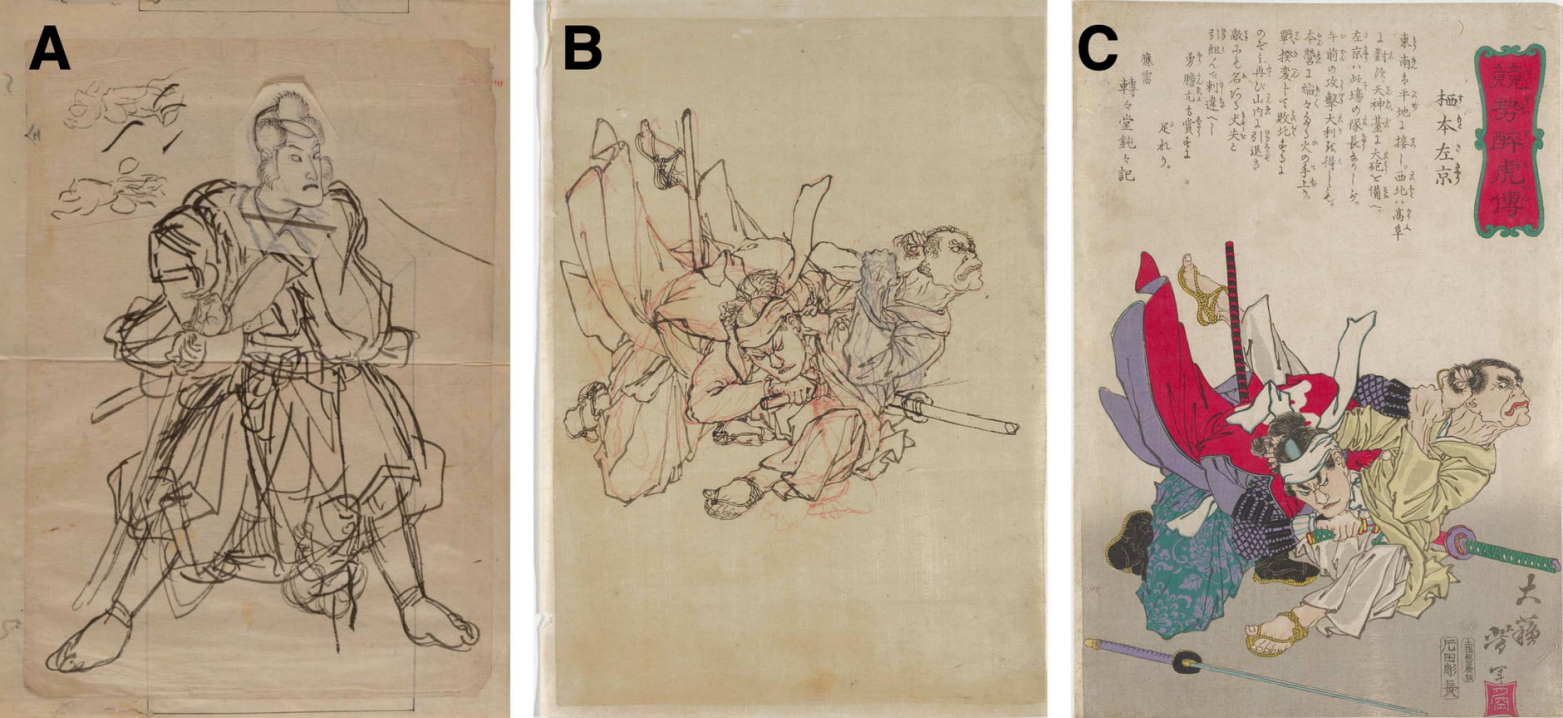
Pilot studies in art. **A** Preliminary study, or shita-e (  “under-drawing”) by Utagawa Kuniyoshi (  ). Note the head between the warrior’s knees, which seems to have been an earlier sketch of the face. The final preparatory design of the face has been drawn on a separate piece of paper, attached to the sketch and pasted over an earlier rougher drawing. Dated between 1840 and 1860. **B** Preliminary study by Tsukioka Yoshitoshi (  ), dated 1874. The *red drawing* illustrates Yoshitoshi’s attempt at repositioning the foot, while the paper placed on the drawing highlights where the hand was re-drawn. **C** Final print of Sumoto Sakyo (  ) by Yoshitoshi, dated 1874. Panel A courtesy of the RISD Museum, Providence, RI, accession number 49.437. Panels B and C courtesy of the Minneapolis Institute of Art, accession numbers 2017.106.120 and 2017.106.119

In art, preliminary studies or sketches may be used to try out many aspects of an artwork, including the overall composition, specific poses, facial expressions, background elements, etc. There are many famous examples available online, including studies for ‘The Last Supper’ by Leonardo da Vinci, for the painting ‘Nighthawks’ by Edward Hopper, or in the work of Rembrandt van Rijn (see, e.g., Royalton-Kisch, 1989).

Figure 2 shows examples of studies in Japanese art from the 19th century. Consider the preliminary study in Fig. 2A. This example by Utagawa Kuniyoshi shows a head drawn between the warrior’s knees, which seems to have been an early sketch of the face, which was subsequently abandoned by turning the paper around. The final design for the face is drawn on a separate piece of paper, which is then pasted onto the design over a rougher, earlier version of the face. This example nicely illustrates the iterative process of design in art: earlier versions of the face were quickly abandoned, or revised by pasting over older versions. Such preliminary sketches could be used to quickly try out various elements of an artwork, e.g., various poses or facial expressions, before commencing the final design. A similar approach may be seen in the preliminary study in Fig. 2B. The red part of the drawing shows Tsukioka Yoshitoshi’s attempt at repositioning the feet in a different manner, while the paper pasted onto the print allowed Yoshitoshi to re-draw the character’s left hand. The final print of this work (Sumoto Sakyo) is depicted in Fig. 2C.

A similar iterative process can be observed in product design, which Norman (2013) describes as a series of phases, including observation, idea generation (ideation), prototyping, and testing. He writes that “This is often called the spiral method ... to emphasize that each iteration through the stages makes progress.” (p. 222). Note the intuitive resemblance to the empirical cycle described above. Again, many famous examples exist. One notable example is the first bagless vacuum cleaner designed by James Dyson. In various interviews, he describes it took over 5000 prototypes or iterations over the course of 15 years, a lot of tweaking and refining, before he had a working product that could be sold. Comparing pictures of earlier prototypes, the first model, and the current state of the art in cyclone vacuum cleaners, shows a beautiful progression of the product through iterative design^3^. Similar progressions can be seen for many other products, including the first and last authors’ favorite folding bike, the Brompton (Butler-Adams & Davies, 2022). Other products are not as successful. One notable example is the Nintendo PlayStation, a console developed by Sony and Nintendo. The Nintendo PlayStation made it to the prototype stage, but it was never commercially pursued^4^. Like in art, product prototypes may be built for very specific purposes, from testing the look and feel of a product, to testing software functionality on the product (see, e.g., prototypes of the first Apple iPhone).

The purpose of product prototypes or preliminary studies in art is clear: they are great for quickly trying out things, failing fast, and revising ideas. Some ideas will be killed (as in the Nintendo PlayStation example), while others may be developed further (as in the Dyson example). Even if ideas are killed, the knowledge gained may be a fruitful source for future studies (as we assume is the case for the preliminary study in Fig. 2A). Translating these principles to empirical science, one can see pilots being used for many different aspects of setting up and conducting an experiment; testing early versions of experiments, procedures or protocols early and often, failing quickly, and avoiding bigger mistakes later^5^. We now turn to what such mistakes in empirical science may look like in the context of eye-tracking research.

## Why do you need pilot experiments?

What might go wrong if one does not carry out proper pilot experiments? The following examples from eye-tracking research are meant to illustrate the breadth of issues one may encounter that pilot experiments may help to alleviate. Some of the examples come from eye-tracking studies that were published in the end, but most come from what we or the participants in our various eye-tracking courses have experienced over the past decade or so.

In going through the examples, it is useful to keep in mind that when we describe pilot experiments, we do not mean just a full experiment run with fewer participants as suggested in, e.g., Cozby and Bates (2015) or in some of the definitions in Thabane et al. (2010, Table 1). Rather, pilot experiments can vary tremendously in scope and purpose (see also Van Teijlingen & Hundley, 2001, Table 1): They can be as small as trying out what font size or line spacing to use in an eye-tracking study on reading, or finding out what chair keeps participants sitting the most still in front of the eye tracker. Depending on the goal of the pilot experiment, it can be conducted with just the experimenters, with colleagues, or with naive participants. A pilot experiment can be any empirical test that brings one toward the final design of a study.

### Reason 1: To find out if you need an eye tracker

Probably the first reason why pilot experiments can be useful, is to find out whether an eye tracker adds anything to a study. In our eye-tracking courses, we often encounter early career researchers who have been given a near-impossible job. Either they have been made responsible for adding eye tracking into a larger project, or tasked to utilize eye-tracking data that has been collected already, in some meaningful way. The problems that these researchers often encounter are that the use of eye tracking is ill-motivated, or that there are no clear expectations of what the eye-tracking data may reveal (see also Godfroid & Hui, 2020).

Two crucial questions one can ask are (1) whether eye movements are needed for executing the behavior of interest, and (2) how eye movements may be related to, e.g., performance, participant states, or other behavior. These topics are addressed in detail in parts 1 (Hessels et al., 2025b) and 2 (Hooge et al., 2025) of the article series. Pilot experiments could help assess the importance of eye movements or gaze behavior in the broader context of a research project, or to find operationalizations of the construct of interest that would provide meaningful scientific insights and thereby justify the use of an eye tracker. For example, we have seen many cases where aspiring eye-tracking researchers have collected hours of wearable eye-tracking data in real-world environments (e.g., classrooms, navy command decks, supermarkets) with little to no idea of how to go about data analysis and interpretation. However, these examples did not end up in the scientific literature, nor are we aware of other examples that ended up being published. Pilot experiments can be crucial in assessing the necessity or added value of an eye tracker prior to wide-scale data collection.

### Reason 2: To test your experiment

A second reason to conduct pilot experiments is to test one’s experiment in a very practical sense. Does the experiment work as expected? Is the stimulus presentation as intended, e.g., with regard to trial orders, presentation times, etc.? Are all relevant data and experimental parameters stored as needed for data analysis?

**Fig. 3:**
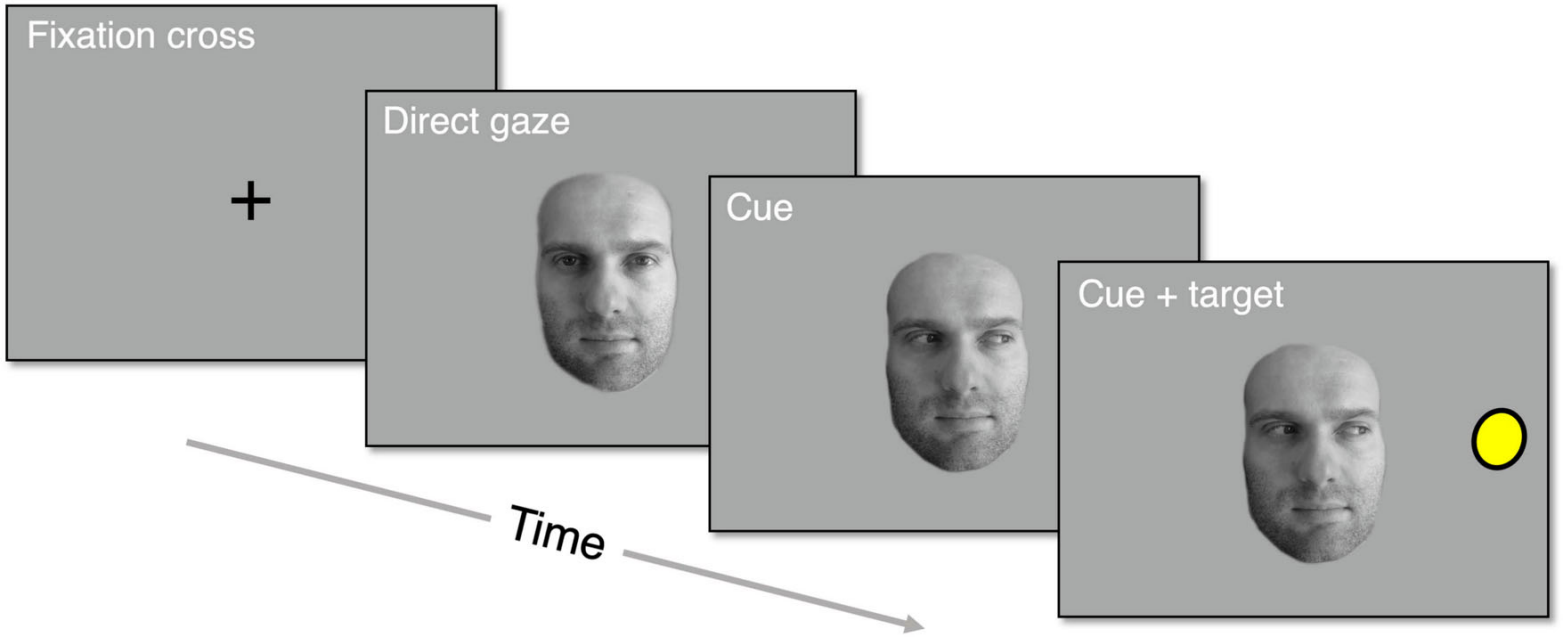
Overview of one example trial in a gaze cueing experiment. Each trial is characterized by the cue direction (face looks left, face looks right), target location (left on screen, right on screen), and thus the cue-target congruency (congruent, incongruent). Additionally, different faces may be used. Responses to the target location can be made manually, e.g., by pressing left or right arrow keys, or by making a saccade to the target. Here, a congruent trial with right-sided cue and right-sided target is depicted

The first author was involved in a large longitudinal study involving eye-tracking experiments conducted with children of various ages (Onland-Moret et al., 2020), working specifically on training research assistants to conduct eye-tracking measurements and data quality control (Hessels & Hooge, 2019). Among the experiments was a gaze-cueing experiment (see, e.g., Frischen et al., 2007). On each trial, a face is presented that either looks in the direction in which a target will appear, or in the opposite direction (see Fig. 3 for an overview of a trial). Each trial is characterized by a cue direction (face looks left, face looks right), target location (left on screen, right on screen), and thus the cue-target congruency (congruent, incongruent). In addition, 10 different face identities were used to depict the gaze cues. All variables should have been perfectly counterbalanced. However, when preparing the data-analysis pipeline, it was discovered that facial identities were, in fact, not perfectly counterbalanced across the various cue-target combinations. That is, not all unique trial combinations occurred, or occurred equally frequently. The error was rectified and the changes to the experiment were recorded in a change log so that any impact on outcome measures could be assessed later on. However, this was after measurement sessions had already been conducted for 1500 participants.

How may this problem have been prevented? When developing the data-analysis pipeline, a number of assumptions about the experiment were explicitly checked: how many trials are presented? How many of each unique combination of cue direction, target location, and facial identity? Upon verifying that the experiment was presented as intended, the outcome measures (e.g., saccadic reaction times) were computed. If the development of the data-analysis pipeline had been done prior to conducting the measurements, and if it had been piloted, the problem may have been prevented. In this case, determining whether the assumptions about the experiment were correct helped to identify the error. Of course, the problem could also have been much more consequential, for example if cue direction, target location, or trial congruency had been incorrectly logged.

As another example of a consequential mistake, one of us made a last-minute change in the code of an eye-tracking experiment, leading to log messages being sent before the logging of the eye tracker’s recording software had started. These log messages were therefore not stored with the eye-tracking data. When trying to match the eye-tracking data to individual visual stimuli (i.e., pictures) later, it was found that no information about what image was shown was stored with the eye-tracking data, making analysis impossible. Obviously, recordings that were already made had to be redone.

The main message is not that mistakes such as the two described here ought not to occur, but rather that such mistakes are bound to happen and that dedicated pilot experiments can help to test assumptions and find mistakes early, so that the impact of potential mistakes is minimal. This applies to testing one’s experiment, testing and improving the eye-tracking setup, as well as the data collection and data analysis.

### Reason 3: To test your data-collection procedure

A third reason for conducting pilot experiments is nicely illustrated by Birmingham et al. (2017). They conducted a study on spontaneous gaze following behavior in 8–16-year-olds with and without autism spectrum disorder (ASD). The children were engaged in several games with an experimenter while they wore a wearable eye tracker (ASL Mobile Eye). In addition, webcams were positioned in the room to record participant and experimenter behavior. The authors write that “Due to unreliable eye-tracking data for the majority of participants, we coded only the HD webcam footage” (p. 246). That is, all eye-tracking data were excluded from the data analysis. The authors explain in more detail why the eye-tracking data was unreliable, attributing this to problems with calibration or loss of calibration after starting the experiment. More specifically, they write:

“If we were unable to calibrate the eye tracker, it was due to fluorescent lighting from the ceiling that created a glare in the reflective lens, interfering with the detection of corneal reflectors; and/or the participant’ facial structure did not support the goggles and the camera did not adequately capture the eye; and/or calibration failed for unknown reasons, possibly due to limitations of this older eye tracking system. [...] Reasons for loss of calibration during the experiment included the following: participant’s facial structure did not adequately support the eye tracking glasses on the face, causing the glasses to slip and lose the image of the pupil; participant accidentally bumped the eye tracking glasses during the experiment, or made facial expressions, or squinted, leading to intermittent loss of signal; glare from overhead lighting interfered with corneal detection ...” (Birmingham et al., 2017, p. 246 )

What might the researchers have learned from pilot experiments? If the data-collection procedure had been tried out with a few representative participants in the same recording location as the one described by the authors, we assume many of the problems highlighted above would have been encountered. Other recording rooms might have been sought out to circumvent the problematic overhead lighting. If the eye trackers had not been sufficient for most or all participants in a pilot experiment, the authors may have either chosen to use different, perhaps more modern, wearable eye trackers, or might have optimized their webcam setup further and bypassed the eye-tracking data collection altogether.

Luckily for the researchers, they had a backup (i.e., the webcam footage), which proved sufficient to answer some of their research questions on spontaneous gaze following in children with and without ASD. Because they did, and because they wrote extensively about the problems during the eye-tracking data collection, we can use this as an example. However, we assume there are many other examples like the present one that are not known to the scientific community, because they do not end up in publications. For example, one of us was involved in an experiment where calibration of the eye tracker was done using red fixation markers on a black background, while the stimulus material during the experiment was much lighter. This resulted in large pupil size changes from calibration to the stimulus material, and subsequent systematic errors that exceeded the size of the Areas of Interest in the eye-tracking data analysis (e.g., Wyatt, 2010; Hooge et al., 2021). Piloting the data-collection procedure and conducting initial data-quality checks could have prevented such a problem. This example also shows that designing an experiment and collecting and analyzing data are intimately related. Postponing potential problems to the data-analysis stage is a risky strategy.

### Reason 4: To design and refine your data analysis

Another reason to run pilot experiments is to design and/or refine one’s data analysis plan. This point relates closely to reason 1 above; if it is clear what the eye tracker adds in a study, a feasible and meaningful operationalization of the construct or behavior of interest can be utilized. When this is not the case, we often see researchers aiming to describe everything. One such example relates to a wearable eye-tracking study with manual mapping of fixations to areas of interest (AOIs). Using the open source GazeCode software (Benjamins et al., 2018), users can manually but quickly assign AOI labels to fixations^6^. The software allows for mapping up to nine AOIs, corresponding with each of the numbers on the numpad. One participant in one of our eye-tracking courses asked the developer of GazeCode whether 81 AOIs were also possible, as he would need that for his analysis. Clearly, when done manually, a staggering 81 AOIs makes for a very time-intensive data analysis, for at least two reasons. First, it may be a difficult decision process for the annotator, which increases the mapping time for each fixation. Second, it may practically take more time to select between 81 options than between nine options. Pilot experiments may, for example, help (1) elucidate which AOIs are not looked at all, removing them from the AOI set, (2) assess the maximum number of AOIs for which feasible data analysis is possible within a reasonable time frame, or (3) determine which AOIs are strictly necessary to test one’s hypothesis. Often, we have found that the research questions or hypotheses are not about 81 (or some other large number of) AOIs, but rather that researchers are worried about ‘missing’ something. Proper piloting can ensure relevant findings in the context of one’s research are not missed, while data analysis is kept feasible.

Another example where piloting may have helped produce a (more) feasible data-analysis pipeline is the wearable eye-tracking study by Maran et al. (2022). They had 118 individuals walk 5–7 min through a crowded shopping mall and then annotated the recordings frame by frame for looks to the faces or bodies of other people. Annotation took 830 h in total (the equivalent of 5 months of full-time work for one annotator, coding 40 h per week), or 7 h per recording. Assuming an average 6-min recording time, this means annotation took 70 times the recording time. Based on the comparison of software (GazeCode vs. Tobii Pro Lab) that allows manual annotation per fixation or slow phase by Benjamins et al. (2018), this might have been achieved much faster. Benjamins et al. (2018) had participants annotate 330 s of wearable eye-tracking data, which took on average 880 s in the GazeCode software (i.e., 2.7 times the recording duration), and 2880 s in Tobii Pro Lab software (8.7 times the recording time). Finally, computer vision techniques might have allowed fully automated analysis of looks to the faces and bodies of other people (see, e.g., Hessels et al., 2020a). Dedicated tests of the data analysis by Maran et al. (2022) could have been used to decide upon a reliable and feasible data-analysis plan.

### Can we not ‘fix’ it afterwards?

From the above examples, it is clear that pilot experiments can help assess the utility of an eye tracker, and test experiments, data-collection procedures, data-analysis pipelines, etc. We see pilots as essential tools to simplify one’s study. However, they require researchers to make informed choices about what to measure, estimate, or analyze, but also what to omit from a study.

One alternative is that researchers do not exclude certain measurements, factors, conditions, or AOIs from a study, but rather feed them as additional variables into one’s statistical analyses. For example, by adding additional AOIs to the statistical model, or variables such as gender, measurement location, or background knowledge of the participant. There are at least three reasons why we think this is not a sensible idea, if these variables and their combinations are not of primary interest. First, the results of a 4x3x5x2 within-subjects MANOVA are much more difficult to interpret and explain than the results from a *t* test with one eye-tracking measure. If one’s research question can be answered with one *t* test rather than more complicated statistical models, we would advise one to do so. Second, as described by Brenner (2016), adding additional factors to one’s statistical analyses and testing them for statistical significance may mean inflating the number of false positives, even for effects that are not of primary interest. Third, some things cannot be ‘fixed’ by complicated statistical analyses, such as floor or ceiling effects. As an example, consider an eye-tracking study on the perception of facial identities or expressions. Several studies have shown that already two fixations may suffice for face recognition (Hsiao & Cottrell, 2008), while happy facial expressions can be recognized as such within 50 ms (Neath & Itier, 2014) or from peripheral vision (Calvo et al., 2014). Adding various presentation times and more/less subtle facial expressions and investigating the relation between the number of fixations and categorization accuracy, may yield ceiling effects for many of the less subtle expressions. Complicated statistical models will not ‘fix’ ceiling effects at a later stage. Of course, in this instance, previous research may give a good idea of the boundaries on recognition or categorization performance, but this need not be the case for every research topic. Pilot experiments can be used for such purposes.

## Pilot experiments: A series of examples

In the present section, we outline the pilot experiments conducted for a series of studies on the role of gaze behavior in collaborative interactions (Hessels et al., 2023, 2024, 2025a). The goal of these elaborate examples is to show how pilot experiments can be effectively utilized, and that they can be used iteratively in various stages of empirical research, for answering many different questions related to one’s research. The present series of studies included multiple participants, wearable eye trackers, a custom measurement setup, and an automated data-analysis pipeline, and may therefore be considered to be particularly ‘difficult’ eye-tracking studies. However, these attributes allow us to make explicit the diversity of the problems and questions that were piloted, and to show what changes were made as a result. The examples already presented above show that pilot experiments are also invaluable for many other (seemingly simpler) eye-tracking studies. Thus, the pilot experiments described in this section are not meant to be taken as a blueprint for all eye-tracking research, but they may serve as inspiration to readers for applying pilot experiments effectively to their own research.

**Fig. 4:**
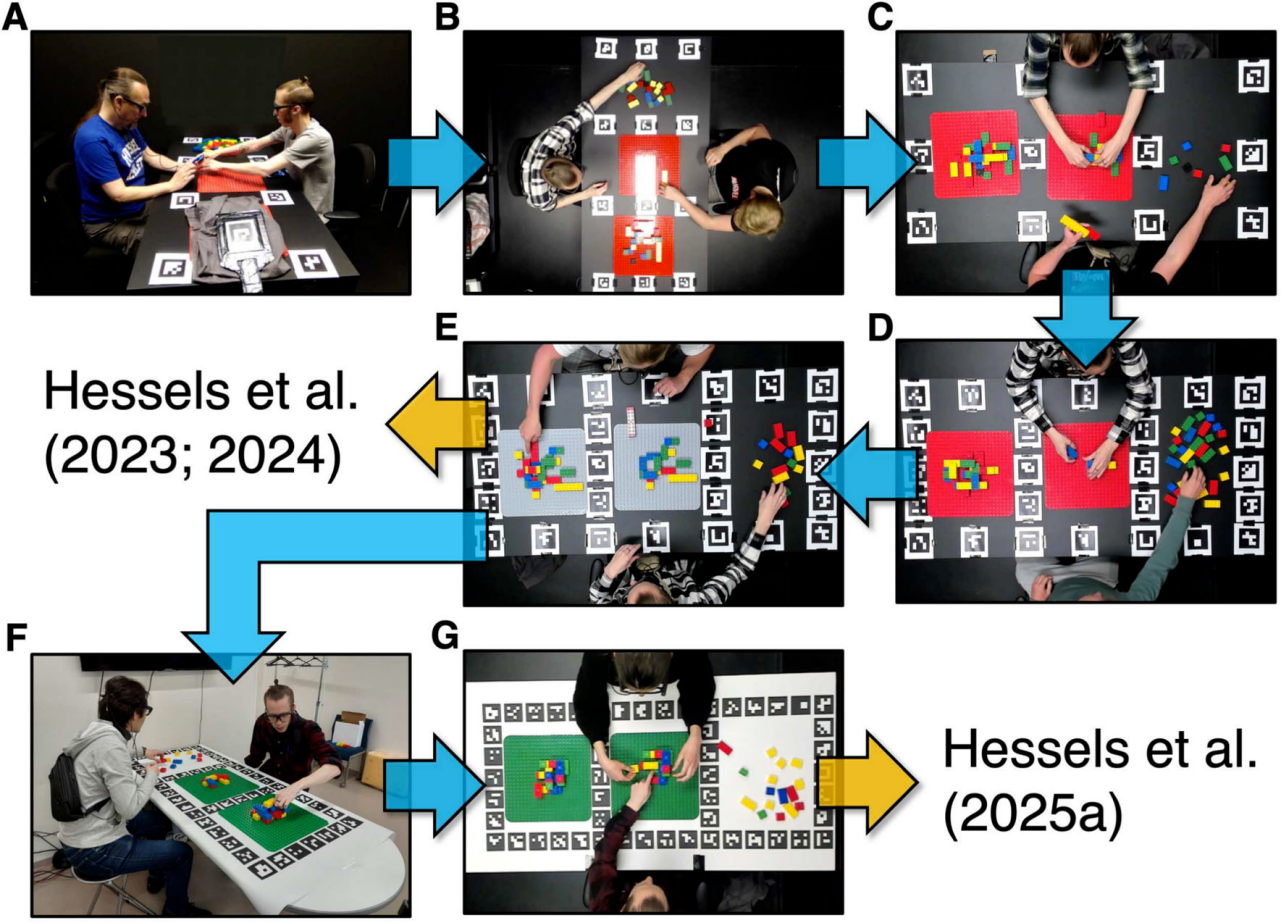
Pilot experiments during setup development. Panels **A**–**G** depict various iterations of the eye-tracking setup for studying gaze behavior during dyadic collaboration used in Hessels et al. (2023, 2024, 2025a). The changes made after each iteration of the setup are described in detail in the main text

### Formulating the scope of the studies

The first author (RH) has been interested in the role of gaze behavior in human interactions for a long time. In a series of studies on building DUPLO models together (Hessels et al., 2023, 2024, 2025a), the interest was specifically on the relation between gaze behavior, (non-)verbal communication, and task-related behavior during collaborative interactions. The studies were conducted with pairs of participants engaged in various collaborative tasks, while their gaze behavior was recorded using wearable eye trackers. The first plan to pursue this series of studies came from a wearable eye tracker test (Hooge et al., 2022), where one eye tracker was found to be particularly quick to set up with a participant. This feature makes it a particularly interesting eye tracker in the context of studies with multiple participants recorded simultaneously: It saves a lot of time or can be done with a single operator, instead of multiple operators.

The first step was to decide on what to study. While the broad theme – gaze in relation to communication and task-related behavior – was clear, the exact research questions and the collaborative interactions to investigate, had yet to be defined. In this context, multiple student research projects were set up and conducted, focusing on refining the research questions, deciding which interactions are interesting and feasible to study. Many practical things about recruitment, participant behavior, etc., were learned in this context. We also uncovered things we did not expect, for example, that during some collaborative interactions, pairs of participants did not communicate verbally or make eye contact at all. Of course, these types of interactions were not pursued further. The second step was to develop the setup. The main requirements were that (1) two participants could interact and collaborate, (2) their gaze behavior could be recorded simultaneously, (3) eye-tracking data analyses could be automated. Before we describe the iterative development of the setup, we recommend the reader to watch some of the example videos available at https://osf.io/2q6f4/ to get an idea of what the setup looked like in practice.

### Development of the physical recording setup

The most explicit results from the pilot experiments can be seen in the development of the physical environment in which the eye-tracking recordings were conducted. Figure 4 depicts various iterations of that recording environment or setup. Panel A depicts a very first try: two participants are seated at either end of a table while wearing Pupil Invisible eye trackers (Tonsen et al., 2020). In front of them on the table are (1) a Lego Duplo model to be copied currently hidden under grey cloth, (2) a Duplo build plate on which the model can be reproduced, and (3) an area with Duplo building material. Printed fiducial markers, specifically ArUco markers (Garrido-Jurado et al., 2014), are placed on the table. These are meant to allow automatic transformation of the gaze location in the eye tracker scene camera video to a location on the table.

Figure 4B depicts the first actual setup from the perspective of the top-view camera. This camera was added to allow analysis of the manual task-related behavior of the participants which may not always be in view of the eye tracker scene cameras, as well as allowing for visualizing the gaze location of both participants in the same view after transformation to the table coordinates (see example video available at https://osf.io/2q6f4/). While both participants and the table are clearly visible in the top-view camera, there are several issues. For example, there are substantial reflections on the table and Duplo build plate from overhead lighting and the fiducial markers are blurry. Furthermore, the aspect ratio of the camera is not used to its full advantage for capturing the wide table. Rather, the long axis of the video camera is rotated 90° with respect to the long axis of the table. This means most of the camera image is used to record a part of the room where nothing happens.

Figure 4C depicts the setup with an improved top-view camera and a modified camera orientation. As can be seen, the reflections from overhead lighting depicted in panel B are no longer present, and the fiducial markers are well in focus. While theoretically there are enough fiducial markers to allow mapping of gaze from the scene camera video coordinates to a location on the table, the participants’ hands or Duplo blocks may sometimes (partially) occlude one or more of these markers in the eye tracker scene camera image. Moreover, participants may orient their head in such a way that only one or two fiducial markers are visible at all in the eye tracker scene camera image. To make the setup more robust, more fiducial markers were placed on the table, as visible in Fig. 4D. In panel E, the red Duplo build plates have been replaced by grey ones. This was done to facilitate automatic analysis of which blocks (i.e., based on color from the video) are picked up and placed on the build plate. In previous versions, red blocks would be placed on red plates, which would be difficult to automatically distinguish. The version of the setup as depicted in panel E was applied in Hessels et al. (2023, 2024).

As a follow-up to Hessels et al. (2023, 2024), a cross-cultural study was planned with Hamamatsu University School of Medicine, Hamamatsu, Japan. This meant reproducing the setup from Utrecht University in Hamamatsu. There, a few additional constraints were placed on the lab space. Specifically, the room available for conducting measurements was also utilized as a meeting room, so the setup needed to be easy to build up and store again after measurements. Various solutions were tried out, resulting in the setup depicted in Fig. 4F. Here, the fiducial markers are no longer pasted on the table, but are printed on a poster that can be rolled up so that the setup can easily be cleaned away. Crucially, the poster contains extra white cloth on one side. If the side with the markers is positioned against the wall, the extra white cloth drapes over the table on the other end, which is not problematic. If the side with the extra white cloth is positioned against the wall, the marker layout drapes over the table on the other end, which means one part of the table marker layout is not actually on the table, making it obvious to the experimenter that this is not the right orientation of the marker poster. Thus, the marker layout can only be placed in one direction on the table, preventing rotated marker layouts from measurement to measurement. An additional change is the color of the Duplo build plates, which are now green. This is for pragmatic reasons, as grey plates were unavailable through suppliers at Hamamatsu University School of Medicine during the visiting fellowship tenure of author RH. For the cross-cultural study, we opted for the same visual layout of the setup in both countries, rather than stick with the grey plates in one country (the Netherlands). The grey plates were initially used to optimize the contrast between the colored blocks and the building plates and to possibly allow automated analysis of which blocks were picked up, but this was not used in our previous two studies, nor crucial to the planned analyses for the cross-cultural study. Thus, when forced to choose between the consistency of the setups and the potential future automation of analyses, we opted for consistency of the setups. Figure 4G depicts the setup in Utrecht, modified to match the changes made in Hamamatsu. These versions of the setups were then applied in Hessels et al. (2025a).

### Refining the setup, data analysis, and experimental protocols

While the development of the physical recording setup can easily be visualized, many more aspects of the studies were piloted and modified. Here we present a non-exhaustive list of these pilot experiments. It is important to realize that these pilot experiments differed tremendously in scope: some were short tests of 5–10 min with just an experimenter, others were more substantive and/or were conducted with naive participants.

#### Eye tracker scene camera settings

What should the exposure setting be for the eye tracker scene cameras? If exposure is short, motion blur in the camera image due to head movement of the participant is less evident than for longer exposures, but the scene camera image is very dark. This is great for ArUco marker detection, but worse for automatic face detection, which seemed to work better for slightly blurred faces than for underexposed faces. As we needed both reliable ArUco marker detection to map gaze to the table coordinate system, and face detection to determine when people look at each other’s face, we used pilot experiments to determine the exposure setting that would strike a good balance between the robustness of the two methods. These pilot experiments involved only the experimenters and recordings were usually very short (< 5 min). Note that robust fiducial marker and face detection under conditions of motion blur are active problems in computer vision (e.g., Li et al., 2019; Kalaitzakis et al., 2021).

#### Camera synchronization

In order to express gaze behavior from both participants recorded with separate eye trackers, and task-related behavior annotated from the top-view camera in the same time, the camera recordings had to be synchronized. At first, we had a naive idea that we only needed one transient that was visible in all three cameras (e.g., a clapperboard) to synchronize them, i.e., to find out the start time of the experiment in each camera recording. However, we wrongly assumed that all three cameras had accurate clocks that would keep running at constant intervals. To find out exactly how accurate and precise the synchronization between the eye trackers and the top-view camera was, we conducted a separate pilot experiment where we presented many synchronization transients spread throughout a long (30+ min) recording. The results from this pilot experiment affected the way synchronization took place as well as informing us about the temporal limits of the analysis. Specifically, we decided to synchronize the recordings by using two synchronization transients instead of one (for details, see Hessels et al., 2025a).

#### Improving the data-analysis pipeline

We conducted many pilot recordings to test the gaze mapping and synchronization procedures, the assessment of data quality, and the assignment of gaze to AOIs (i.e., locations on the table, and the face of the other person one interacts with). A few concrete problems and solutions were encountered. First, a face-detection method we had been using for a long time (OpenFace, see Baltrušaitis et al., 2016) was not reliable enough for our eye tracker scene camera videos. We therefore opted for the more modern YuNet face detection model (Wu et al., 2023). Second, the procedure for synchronizing eye trackers with their scene cameras and the method for estimating accuracy and precision (specifically the GlassesValidator procedure, see Niehorster et al., 2024a) were conducted separately, which required separate analysis pipelines and took too much time. These were therefore integrated into one procedure. Third, smaller issues with synchronization or mapping gaze to the table were encountered, which led to updates to the analysis software (gazeMapper, see Niehorster et al., 2025).

#### Camera placement

At Hamamatsu University School of Medicine, we could not attach a video camera to the ceiling. We therefore piloted the best placement of the video camera that would allow a similar top-down view as in Utrecht, from which participants’ behaviors of interest could be annotated. Experimenters and colleagues acted as participants to ensure representative recordings.

#### Miscellaneous aspects of the setup

Seemingly mundane aspects of the setup were piloted: what kind of chairs or stools should we use? How long can we make recordings for? How long do the batteries on the eye tracker recording units or attached phones last? How do we position the recording units to prevent them from overheating or being in participants’ way? These pilots often led to minor modifications in how the experiment was conducted. Many of these pilot experimenters were conducted with just the experimenters or with interested student volunteers or colleagues.

#### Protocol development and refinement

Many aspects of the experimental protocol were piloted. Which Duplo models should be used such that collaboration takes place between the participants? How do we ensure that the Duplo models can be adequately prepared by the experimenters? How should the participants be instructed to prevent misunderstanding? What information do we present about the eye trackers or goal of the study before the recordings such that participants are not made aware of our hypotheses (cf. Orne, 1962; Cook et al., 1970)? How do we ensure the same instruction at Utrecht University and at Hamamatsu University School of Medicine such that participants execute the task in the same general manner? These questions were discussed at length and tested in various, often brief, pilot experiments. When needed, naive participants were invited to take part in the pilot experiment.

The varied nature of the pilot experiments outlined above and the modifications they led to illustrate that decisions on many aspects of the recording setup, experiment, and even the exact research questions cannot be made behind a desk. We have been surprised more often than not during our pilot experiments, finding that devices or participants behave differently than expected, while issues we worried might crop up did not materialize at all.

## Practical advice on piloting

So far, we have presented the relevance of a piloting mindset, the problems one can encounter when proper pilot experiments are not conducted, and a series of practical examples in the context of eye-tracking research. To conclude, we present practical advice that may aid readers when setting up their own eye-tracking studies. The advice is organized around five themes, (1) take enough time, (2) be problem-oriented, (3) pilots are of an iterative nature, (4) many questions are empirical, and (5) apply the four-eyes principle.

The advice to “take enough time” may seem rather self-evident. But how long does piloting take, and what exactly is enough time? As outlined at the very start, de Groot (1961, section 5;1;4, p. 146) suggests “... as a rough estimate – about a quarter of the time”. We are doubtful whether such a rule of thumb would be applicable to all empirical eye-tracking research. For example, we have conducted multiple wearable eye-tracking studies where the piloting and preparation phase took 6–9 months, while the measurements were conducted within one afternoon (Hessels et al., 2020a, b). The reason was that we needed many people to be present at the same time to serve as the research participants and as human crowds. In such scenarios, the stakes are high: If anything goes wrong on the measurement day, another measurement day with 40–60 people needs to be scheduled. Thus, we planned and piloted for many contingencies. Luckily, as one of our eye trackers malfunctioned on a measurement day and we needed to use our backup eye tracker. At the other extreme, studies may run for 10 years or more (as in the example cohort study presented above, see Onland-Moret et al., 2020). In that example, even a 1–2 year piloting phase does not reach the estimated quarter of the time. What is more important is that experiments, signal processing or data analysis pipelines, and/or participant recruitment and instructions have been carefully tested and seem to function as expected. In some cases, this will take more time than in others.

Practically speaking, we urge researchers to be careful if they hear statements like “we have no time for piloting and testing, we should have started with the official recording sessions last week”. We have often heard this, especially in the context of (too) ambitious grant projects. In our experience, not taking the time to carefully conduct pilot experiments is actually more costly in terms of time and money, as careful piloting can prevent problems that take much longer to correct afterwards, if possible at all (as illustrated in some of the examples above).

Second, we advise researchers to adopt a problem-oriented attitude rather than a solution-oriented attitude when planning and setting up an eye-tracking study. In our experience, an emphasis on quick solutions may yield solutions that underestimate the problem or are not scalable in the long run, e.g., because they require manual tweaks or time-intensive labor. Additionally, quick fixes may have repercussions down the line that cannot be properly anticipated. A problem-oriented attitude means trying to understand the problem at a deeper level before deciding on a solution, as in our earlier examples when deciding on the eye tracker scene camera settings or the camera synchronization. Again, we urge researchers to be careful if they hear “we will fix that afterwards”. As illustrated above, many issues simply cannot be fixed afterwards. Underestimating problems when they arise and overestimating oneself may lead to a host of problems later on.

Third, pilot experiments are of an iterative nature. Each iteration may take more or less time, and have more or less impact on the future design of the study. This has very practical implications, for example in the context of medical ethics committees or institutional review boards, as well as in the context of pre-registration (a point also addressed in the context of empirical research in Hessels & Hooge, 2021). If such committees require a research protocol to be approved for any empirical research, no matter how small in scope, proper piloting may quickly become infeasible. For example, we have encountered situations where the researchers had to apply for ethics approval to pilot eye trackers on themselves. This took 4–6 weeks to be approved by the ethics committee. In the context of the series of pilot experiments presented above, this procedure would have easily led to a 2+ years period of piloting. In our view, a proper understanding of empirical research and pilot experiments is essential for ethics committees to function efficiently and not harm science in the process. For researchers, this means understanding the governance within which one has to operate, to conduct effective and efficient (pilot) experiments.

Fourth, we advise researchers to keep in mind that many questions are empirical in nature. As mentioned, we have been surprised more often than not during our pilot experiments about the outcomes. Yet, we have also often heard people tackle discussions about empirical matters with phrases such as “I can imagine that so and so will be the case”. Such imagination is a thorn in the side of empirical research. In our experience, armchair philosophy is by far not capable of capturing all the things that may happen, nor predicting reliably the outcome of even simple investigations. If a question is empirical in nature, and it is within one’s means (time, equipment, money, etc.), testing it directly is more productive. Sometimes the exact measurement context cannot be reproduced, or unforeseen circumstances may occur even after substantial piloting (see, e.g., the section “Practical considerations” in Hessels et al., 2022, for an example of an experiment at a popular science festival). However, proper piloting puts one at an advantage for completing a successful eye-tracking study.

Fifth, we advise researchers to go through pilot experiments together with experts, colleagues, and/or students. We call this the four-eyes principle. Not only do four eyes generally see more than two eyes, piloting together often forces one to make assumptions or expectations explicit, for example, when explaining one’s reasoning to the other party. Asking experts for advice regarding specific aspects of one’s study may additionally be a quick way to identify common problems or to find out which solutions may not apply to one’s research problem.

Finally, in our view, conducting pilot experiments is one of the most enjoyable aspects of the job, especially when one realizes that pilot experiments may save more time than they cost and may result in fruitful ideas for future studies. Empirical science is a very practical endeavor, and practice helps attain high-quality empirical studies. We hope the present article helps students and early career researchers discover, and more established researchers rediscover, the utility and enjoyment of pilot experiments.

## Data Availability

Not applicable.

## Footnotes

1 As an example, the information available to students in the Psychology Bachelor at Utrecht University about their final research project states that “Conducting research is something that you have to learn. This is best learned in practice. This means independently and under supervision *going through an empirical cycle* in psychological research.”

2 In the original Dutch: “... voor goed sociaal-wetenschappelijk onderzoek – als ruwe schatting – circa een kwart van de tijd, inspanning en het budget gaat zitten in de voorbereiding van de (toetsings-)opzet.”

3 See, e.g., https://wiw.hk/magazine/topics/career/how-james-dyson-invented-and-founded-dyson and https://nymag.com/vindicated/2016/11/james-dyson-on-5-126-vacuums-that-didnt-work-and-1-that-did.html accessed 14-01-2025.

4 See, e.g., https://playstation.fandom.com/wiki/Nintendo_PlayStation accessed 14-01-2025.

5 In fact, one could rewrite the first two paragraphs on ‘Iteration’ in Norman (2013, p. 229) easily to fit empirical science: “The role of iteration in human-centered design is to enable continual refinement and enhancement. The goal is rapid prototyping and testing, or in the words of David Kelly, Stanford professor and cofounder of the design firm IDEO, “Fail frequently, fail fast.” Many rational executives (and government officials) never quite understand this aspect of the design process. Why would you want to fail? They seem to think that all that is necessary is to determine the requirements, then build to those requirements. Tests, they believe, are only necessary to ensure that the requirements are met. It is this philosophy that leads to so many unusable systems. Deliberate tests and modifications make things better. Failures are to be encouraged–actually, they shouldn’t be called failures: they should be thought of as learning experiences. If everything works perfectly, little is learned. Learning occurs when there are difficulties.”

6 More specifically, slow phases in the eye-tracking data, see, e.g., Hessels et al. (2018) for an elaboration of the terms ‘fixation’ and ‘slow phase’ in the context of wearable eye-tracking data analysis.

## Declarations

### Open Practices Statement

There are no data or code associated with the present article.
